# Generative Language Models and Open Notes: Exploring the Promise and Limitations

**DOI:** 10.2196/51183

**Published:** 2024-01-04

**Authors:** Charlotte Blease, John Torous, Brian McMillan, Maria Hägglund, Kenneth D Mandl

**Affiliations:** 1 Department of Women's and Children's Health Uppsala University Uppsala Sweden; 2 Digital Psychiatry Department of Psychiatry Beth Israel Deaconess Medical Center Boston, MA United States; 3 Centre for Primary Care and Health Services Research University of Manchester Manchester United Kingdom; 4 Medtech Science & Innovation Centre Uppsala University Hospital Uppsala Sweden; 5 Computational Health Informatics Program Boston Children's Hospital Harvard Medical School Boston, MA United States

**Keywords:** ChatGPT, generative language models, large language models, medical education, Open Notes, online record access, patient-centered care, empathy, language model, online record access, documentation, communication tool, clinical documentation

## Abstract

Patients’ online record access (ORA) is growing worldwide. In some countries, including the United States and Sweden, access is advanced with patients obtaining rapid access to their full records on the web including laboratory and test results, lists of prescribed medications, vaccinations, and even the very narrative reports written by clinicians (the latter, commonly referred to as “open notes”). In the United States, patient’s ORA is also available in a downloadable form for use with other apps. While survey studies have shown that some patients report many benefits from ORA, there remain challenges with implementation around writing clinical documentation that patients may now read. With ORA, the functionality of the record is evolving; it is no longer only an aide memoire for doctors but also a communication tool for patients. Studies suggest that clinicians are changing how they write documentation, inviting worries about accuracy and completeness. Other concerns include work burdens; while few objective studies have examined the impact of ORA on workload, some research suggests that clinicians are spending more time writing notes and answering queries related to patients’ records. Aimed at addressing some of these concerns, clinician and patient education strategies have been proposed. In this viewpoint paper, we explore these approaches and suggest another longer-term strategy: the use of generative artificial intelligence (AI) to support clinicians in documenting narrative summaries that patients will find easier to understand. Applied to narrative clinical documentation, we suggest that such approaches may significantly help preserve the accuracy of notes, strengthen writing clarity and signals of empathy and patient-centered care, and serve as a buffer against documentation work burdens. However, we also consider the current risks associated with existing generative AI. We emphasize that for this innovation to play a key role in ORA, the cocreation of clinical notes will be imperative. We also caution that clinicians will need to be supported in how to work alongside generative AI to optimize its considerable potential.

## Introduction

Patient online record access (ORA) is growing globally [1]. Access includes test and laboratory results, secondary or hospital care letters, lists of prescribed medications, and the narrative reports written by clinicians after visits (the latter referred to as “open notes”). Already, patients across an estimated 30 countries can access some of their records via secure web portals including health apps. In some countries, this innovation is advanced [1]. Since 2021, the federally enacted 21st Century Cures Act in the United States mandated that providers offer all patients access to download their electronic health records without charge [2]. In the Nordic countries, ORA has been implemented incrementally, starting around 2010 [3]. The Finnish patient portal OmaKanta was rolled out with stepwise implementation of functionality between 2010 and 2015 [4]. Patients in Sweden first obtained ORA in one of 21 regions in 2012 [5] with nationwide implementation achieved by 2018. Implementation in Norway began in 2015, reaching patients in 3 out of 4 regions by 2019 [6]. In England, from October 31, 2023, it is mandatory for general practitioners to offer ORA to their adult patients, albeit on a prospective basis [7].

Patients with access to their records report using them to become more involved in their care, to follow up on doctors’ visits, and to obtain an overview of their test results and treatment history [3,8,9]. Multiple surveys show that patients using ORA are positive about the experience after reading their notes. They report many benefits including understanding their care plans better [9], improved communication with and greater trust in their provider [10], and feeling more in control of their health and care [6,8], including doing a better job taking their medications [11,12].

Despite the patient benefits with ORA, challenges with their implementation in clinical practice remain. In this viewpoint paper, we identify outstanding concerns with ORA, which encompass a range of unintended consequences for clinician work burdens, and for the substantial task of conveying bespoke, compassionate, and understandable information to each unique patient who accesses their records. Currently, it has been proposed that a range of targeted patient training and medical education strategies may be sufficient to resolve at least some of these challenges [13-17]. We believe that such interventions are valuable; however, in this viewpoint paper, we explain why the ambitions of such training interventions may be limited.

As a solution, we explain why the use of generative artificial intelligence (AI) may offer more tangible long-term promise than clinician training alone in helping to resolve problems with ORA implementation. While generative AI itself is not new, recent technical advances and the increased accessibility of large language models (LLMs; GPT-4 by OpenAI, LLaMA by Meta, and PaLM2 by Google) have made clinical use increasingly feasible. LLMs are an application of generative AI technology, often defined as machine learning algorithms that can recognize, summarize, and generate content based on training on large data sets. Unlike search engines, which offer pages of internet links in response to typed queries, generative LLMs such as GPT-4 simulate well-reasoned answers couched as conversations. In addition, these models can “remember” previous prompts, helping to build up the perception of dialogic exchange. We review the strengths and limitations of generative AI and emphasize that for this innovation to play a key role in ORA, it will be imperative for humans to be involved as overseers of computer input.

## Current Challenges With Open Notes

### Evolving Functionality of Records

Guidelines, such as those issued by the British General Medical Council, state that clinicians should keep clear, accurate, contemporaneous records that include “...any minor concerns, and the details of any action you have taken, information you have shared and decisions you have made relating to those concerns” [18]. In the era of ORA, clinicians will also need to consider if what they write will be understandable, accessible, and supportive for patients [19]. With the knowledge that patients will read what they write, the functionality of the record is evolving, and this incurs changes with respect to how clinical information is documented [20,21]. Clinicians must uphold the original functionality of the record—documenting the patient’s medical information in clinical detail, but also communicating this information to the patient. With respect to the latter function, it is argued that for records to be understandable and acceptable to a lay audience, clinicians should ideally remove or explain medical acronyms, omit medical vernacular that may be perceived as offensive (such as “patient denies” or “patient complains of”), and strive to convey information in a manner that it is straightforward, comprehensive, and empathic in tone [14]. This is not an easy undertaking for clinicians tasked with pitching information at a literacy level that accommodates diverse patient populations while maintaining the clinical utility of records and adequately serving their medicolegal functions. Indeed, whether such dual functionality is even possible has been questioned [22].

### Documentation Changes

To date, it is unclear whether ORA diminishes the clinical value of documentation [19,23]. However, there is evidence that clinicians may be undermining the accuracy or completeness (or both) of their records, perhaps in attempts to reduce patient anxieties, minimize follow-up contact, or reduce the likelihood of potential complaints [24,25]. For example, in the largest study conducted on clinicians’ experiences of open notes, a 3-center study at 3 diverse health systems in the United States (1628 of 6054, 27% clinicians responded), DesRoches et al [26] found that around 1 in 4 physicians admitted that they changed how they wrote differential diagnoses (23%, n=176), though the nature of these changes is not understood. More worryingly, more than 1 in 5 physicians (22%, n=168) believed that their notes were now less valuable for other clinicians [26].

Conceivably, other changes following implementation of ORA might be more positive. In the study by DesRoches et al [26], 22% (n=166) of physicians reported changes to the use of a partnering language, and 18% (n=139) of them reported changes to how they used medical jargon or acronyms. However, it remains unknown whether such changes improve the comprehensibility of clinical records among patients or whether amendments come with a trade-off in terms of documentation quality.

With ORA, there is also the potential for notes to convey bias of stigmatizing language. For example, in the United States, recent linguistic analysis studies have shown that negative patient descriptors in notes are considerably more common for non-Hispanic black patients and for patients with diabetes, those with substance use disorders, and those with chronic pain [27,28]. It is unclear whether with the knowledge patients may now read what they write, the use of stigmatizing language among these patient populations is being effectively omitted and “cleaned up” by clinicians.

### Work Burdens

Time spent on documentation and patient portal messages remains a growing cause of clinician dissatisfaction and burnout [29]. The impact is exacerbated for clinicians with lower levels of digital competencies, and this “technostress” has been found to directly correlate with burnout [30]. Even tech-savvy young resident physicians have reported the use of the electronic health record as a leading cause of burnout [31]. In the United States, the study by DesRoches et al [26] on clinicians’ experiences, 37% (n=292) of physicians reported spending more time writing notes after patient access was enabled.

Few studies have explored objective measures of the impact of ORA, however, where these measures have been implemented, some of them signal potential for increased patient contact. For example, Mold et al [32] found that the provision of ORA in primary care settings resulted in a moderate increase in email traffic from patients, with no change in telephone contact and variable changes to face-to-face contact. A recent Canadian study found that registration with a primary care web-based portal was associated with an increase in the number of visits to physicians, calls to practice triage nurses, and an increase in clerical workload [33]. Another recent study at an academic medical center in the United States reported a doubling in the number of messages sent by patients within 6 hours after ORA was implemented [34]. It seems reasonable to postulate that at least some of this increased contact may be driven by patients who desire clarifications about diagnoses, results, or other information that is documented in their records.

## Currently Proposed Solutions

To encourage confidence with ORA and to overcome some of these challenges, targeted educational programs have been proposed. Among them are short lists of tips and advice to clinicians, and brief web-based training interventions [13,14,24]. More recently, some medical schools have taken this further. For example, Harvard Medical School has embedded within its curriculum practical training in how to write notes that patients will read [16], and similar work is underway in England [35]. The expressed aim of such training programs is to support physicians in writing notes efficiently and clearly, preserving the necessary clinical details. These programs also encourage students and clinicians to write sensitively and empathically, removing loaded jargon or acronyms that may be perceived as offensive (eg, “follow-up” instead of “F/U,” or “shortness of breath” instead of “SOB”) [14,16]. Notably, however, calls for curricular adaptations are isolated, perhaps reflecting wider uncertainty about ORA among the medical community and the perception that the innovation has been foisted on them.

Similarly, interventions to advise patients about how to engage with ORA appear limited [14,36]. This may be owed to a fear among clinicians that encouraging access to web-based records may exacerbate patient anxiety, lead to increased contact time, or risk disagreements and requests to change documentation. We observe that current recommendations in the published and gray literature offer advice on the benefits and risks of accessing ORA, how to maintain password or portal security, and how to discuss errors or disagreements in their notes with clinicians [14,36].

Combined, these clinician and patient support strategies are valuable but have inherent limitations. Training interventions may be variously implemented and take time to become established in mainstream medical education. Even beyond mainstream inclusion of training in medical curricula, it will also be necessary to target the so-called “hidden curriculum”—the set of unspoken and implicit rules and values that trainees may pick up from their mentors and colleagues within clinical practice [37]. It is unclear whether even those strategies that attempt to convert senior or experienced doctors to the cause are sufficient to counter the hidden curriculum or to neutralize the formation of documentation habits that may not be in keeping with the ORA mandate whereupon clinical notes may now be read by patients and caregivers.

Other recommendations that clinicians should remove all acronyms and medical jargon may present practical dilemmas for upholding the quality of documentation. Aside from extra time spent typing documentation, the capacity to shift from expert to patient perspectives poses unappreciated difficulties. Undoubtedly, many clinicians, as domain experts, might not always fully appreciate when they are using specialist or technical language, nor do they have the attendant skills to convey what they know to patients in an understandable way—a cluster of problems collectively referred to as “the curse of expertise” [38]. Using imprecise language may also have future medical consequences and might result in harm if later clinicians misinterpret what was written [39].

Relatedly, it seems a significant request that clinicians write notes that are bespoke for every patient’s level of health literacy. Yet, each person who attends a clinical visit will have specific health literacy needs. We suspect that the trade-off may lead to clinicians writing notes that are more suited to a readership like them—individuals with higher health literacy and more years of formal education.

Similarly, while often considered a “soft skill,” the adoption of empathetic, encouraging, and supportive language might be a taller order than is frequently assumed. For example, psychologists report that negative biases can curb expressions of empathy [40-44]. Studies show that empathy can be influenced by patients’ race or ethnicity and may be diminished among people presenting with disabilities or already stigmatized conditions [40-44]. Making matters worse, self-inspection may be a particularly weak tool for clinicians to excavate and monitor their own prejudices [45]. Furthermore, the demand that clinicians tailor their notes in ways that are optimized to every patient’s understanding and their emotional needs may lead to not only increased workload but also higher risk of burnout [46].

So far, no objective measures have assessed whether targeted training strategies are effective at improving clinical documentation in terms of preserving medical detail and utility, strengthening patient understanding and patients’ perceptions of clinician support and empathy. We emphasize that while commonly used in training evaluation, self-report surveys will not be sufficient to establish whether educational interventions work in terms of both preserving the detail in clinical notes and supporting patient understanding.

Finally, perhaps most crucial of all, and as already noted, it is unclear whether narrative notes can ever uphold a genuine dual functionality targeting the needs of both clinician and patient readerships [22]. Conceivably, both needs are incommensurable and there will always be a trade-off in detail and understanding should the patient, or the clinician, be given primacy as target reader.

## Generative Language Models Writing Clinical Notes

### Strengths of Generative AI

Doctors strongly desire support with documentation including note writing with surveys showing that they forecast a role for AI in assisting in these tasks [47,48]. Because of their promise with respect to administrative and documentation tasks in health care contexts, LLMs have been described as “the ultimate paperwork shredder” [49]. Owing to the sheer speed and scope of information upon which they draw, LLMs hold considerable potential in generating up-to-date, comprehensive clinical information for patients [50]. This makes the approach particularly promising in generating detailed narrative explanations and summaries of visit encounters. This may help to reduce work burdens on physicians tasked with writing clinical notes.

Another striking strength of LLMs is their capacity to write responses in a requested style or by adopting a specific tone or conversational emphasis. This makes LLMs particularly promising in assisting with writing notes that omit the use of medical jargon or acronyms that are suitable for patients with different levels of health literacy, or among speakers of languages that differ from their provider’s language. This capacity may also help avoid the extra burdens on clinicians attempting to document notes that are tailored to the highly diverse range of unique patient readers.

Preliminary research also suggests that LLMs may help with writing consistently sensitive or empathic notes. In 2023, a highly publicized study suggested that ChatGPT may have better bedside manners than actual human doctors [51]. A team compared written responses of doctors and ChatGPT offered to patients’ real-world health queries using Reddit’s AskDocs forum, where nearly half a million people post their medical problems and verified and credentialed clinicians offer suggestions. On average, ChatGPT responses were 4 times longer than doctors’ replies. A panel of health care professionals—blinded to who or what did the writing—preferred ChatGPT’s responses nearly 80% of the time. The panel ranked chatbot answers as being of significantly higher quality than web-based posts reportedly from doctors; they also judged these reported web-based doctors’ answers as more unacceptable responses to patients. ChatGPT’s responses were rated as “good” or “very good” nearly 4 times more often than those written by the reported web-based doctors, and ChatGPT’s responses were rated as almost 10 times more empathic than those by the reported web-based doctors. At the other end of the scale, these web-based physicians’ replies were perceived to lack empathy approximately 5 times more often than responses produced by ChatGPT.

### Limitations of Generative AI

Despite their potential, LLMs have multiple limitations. The nature of the data sets the models are trained on is critical, as it will determine the scope and nature of responses possible. Of special relevance here, none of the easily accessible LLMs have yet been trained on medical texts and thus lack the core substrate to generate the most appropriate responses. Any bias in the source the models are trained on will also be reflected in answers or text provided. Thus, while a study in March 2023 showed that ChatGPT (version 3) Could pass the United States Medical Licensing Examination [52], the authors of the study noted that to truly assess the potential of such LLMs, there is a need for “controlled and real-world learning scenarios with students across the engagement and knowledge spectrum.” Still, the results of that study were acknowledged by the American Medical Association, which noted that it intends to begin considering how tools such as ChatGPT need to be incorporated into the education process [53].

Indeed, the full extent to which LLMs embed discriminatory biases has not been fully explored. However, it would be surprising if these models did not replicate many of the same biases that already exist in clinical research, and consequently medical education, in part because of the underrecruitment of women, racial and ethnic minorities, and older people. Such skewing is already recognized as a source of disparity with the potential to perpetuate errors or misjudgments in clinical decisions [54-58]. Studies suggest that gender and racial biases are indeed coded into LLMs [59]. It remains unknown whether the potential for such discriminatory errors might prove worse than today with standard human-mediated care; however, some preliminary research suggests that negative stereotyping may be compounded by LLMs [60].

Another concern is the lack of consistency in responses proffered by LLMs. Inputting the same question to GPT-4, for example, rarely elicits the same response. Of course, human responses are rarely consistent as well; however, the extent to which generative AI, relying on LLMs, offers the same level of reliable outputs is uncertain. This is a particular concern given that LLMs are prone to yield falsehoods—a phenomenon referred to as “hallucination.” Moreover, the persuasive conversational tone of LLMs such as GPT-4 means that narrative responses may appear compelling but factually incorrect.

The extent to which doctors may already be adopting generative AI tools, such as OpenAI’s ChatGPT, is not yet known. In the United States, under the 1996 Health Insurance Portability and Accountability Act (HIPAA), which established national standards in the United States to protect patients’ health information from being shared by “covered entities”—that is, providers—to other third parties. Therefore, the use of OpenAI, for example, is precluded under the HIPAA. At the time of writing, in the most common use cases, uploading patient details to versions of generative AI would breach patient trust and medical confidentiality due to privacy concerns.

However, the scope for this is quickly changing. Epic—the US software giant which has an estimated 78% of the share of hospital medical record use in the United States [61]—is currently piloting the integration of HIPAA-compliant GPT services [62]. In addition, an Azure HIPAA–compliant GPT-4 service already exists [63]. Voice-to-text clinical note generation products now represent a growing space in health care. For example, a new app called Ambient Experience from Nuance can listen to the physician’s conversation and, using ChatGPT (version 4), help create the clinical note that is ready for physicians to review [64]. In the United States, such capacities are set to become embedded into electronic health systems, signaling revolutionary changes in medical documentation practices.

### Clinicians and Computers as Coauthors

Combined, the aforementioned discourse suggests that LLMs are far from ready to disintermediate clinicians when it comes to writing clinical notes. We argue that the innovation will play a key role if humans are involved. Thus, this promise could be harnessed if clinicians oversee the cocreation of clinical documentation. In this scenario, LLMs might offer initial draft documentation, which, crucially, should be supervised, and edited by clinicians whose key role in documentation will be to keep a check and balance on the current limitations with these models.

Considering the scope of generative AI, we therefore propose that current training interventions might be constructively adapted to better prepare clinicians to oversee the writing of patient-facing clinical documentation, for example, by editing and checking the quality of clinical information constructed by generative AI and reviewing the sensitivity of the language used. Preliminary studies already show that when humans collaborate with LLMs to coproduce replies to patients, this can enhance patients’ ratings of levels of empathy compared with human-only produced responses [65]. Such partnership could offer a more robust and safe form of documentation quality control—one that could potentially avoid the work burdens associated with documentation burdens and, therefore, the potential for burnout from ORA. We emphasize, however, that training should reinforce the importance of using generative AI as an assistant narrative scribe and not as a substitute for writing notes.

Furthermore, if health systems adopt this approach, we suggest that 2 (or even multiple) versions of clinical documentation may be feasible. Using LLMs, there is scope to not only a complete medical narrative pitched at the level of the domain expert or specialist, but also to document notes couched at the level of health literacy, language, and empathy of the individual patient who might be reading them. This could help overcome the current dilemma of documenting information in a way that is accessible for patients, but which does not diminish the clinical detail for health professionals.

### Future Research Directions

Many research questions could usefully explore generative AI in cowriting clinical notes, especially dual-purpose documentation for both patients and clinicians. We suggest a few novel directions. First, qualitative studies could usefully explore how successfully generative AI translates clinical documentation into patient-friendly language. For example, studies could examine the accuracy and fidelity of generative AI in translating acronyms or other medical jargon, as well as the understandability of the notes, and the level of empathy embedded in patient-facing documentation. Second, experimental studies could probe whether documentation embeds biases or a higher likelihood of containing stigmatizing language for different patient demographics or health conditions. Third, pilot studies could help determine the satisfaction and administrative work burden of dual documentation among clinicians.

## Conclusions

Generative AI is ready for mass use when it comes to writing or cowriting clinical notes, and its potential is enormous. We emphasize, however, that there remain evidence-based risks associated with existing generative AI, which relate to inconsistencies, errors, and hallucinations and the real potential to embed harmful biases in documentation. If carefully implemented, in the long term, doctors who write documentation using generative AI may do a better job of adapting to the evolving functionality of the electronic records than doctors who do not. This adoption may address the potential risk of “dumbing down” clinical documentation while conveying understandable and empathetic information to patients using plain and sensitive language. We also forecast that doctors who cowrite their documentation with LLMs will experience fewer work burdens.

## Abbreviations

AI: artificial intelligence
HIPAA: Health Insurance Portability and Accountability Act
LLM: large language model
ORA: online record access
